# Inflammation mechanism and anti-inflammatory therapy of dry eye

**DOI:** 10.3389/fmed.2024.1307682

**Published:** 2024-02-14

**Authors:** Liyuan Chu, Caiming Wang, Hongyan Zhou

**Affiliations:** Department of Ophthalmology, China–Japan Union Hospital of Jilin University, Changchun, China

**Keywords:** dry eye, inflammation, mechanism, therapy, review

## Abstract

Dry eye is a widespread chronic inflammatory disease that causes fatigue, tingling, burning, and other symptoms. Dry eye is attributed to rheumatic diseases, diabetes, hormone disorders, and contact lenses, which activate inflammatory pathways: mitogen-activated protein kinases (MAPK) and nuclear factor-B (NF-κB), promote macrophage inflammatory cell and T cell activation, and inflammation factors. Clinicians use a combination of anti-inflammatory drugs to manage different symptoms of dry eye; some of these anti-inflammatory drugs are being developed. This review introduces the dry eye inflammation mechanisms and the involved inflammatory factors. We also elucidate the anti-inflammatory drug mechanism and the detection limits.

## 1 Introduction

Dry eye is a prevalent ocular surface disease worldwide, accounting for 11.3% of people over 50, and adversely affects the visual quality, work, and life of patients (1). Dry eye is a multifactorial disease involving conjunctival goblet cells, lacrimal glands, and meibomian glands. These structures affect electrolytes, water, mucin, and lipids in the tear film, causing instability, hypertonicity, and inflammation (2). The tear film, divided into two layers, has the functions of lubrication, antibacterial, nutrition, and wound healing. The inner layer close to the cornea is the aqueous mucus layer, and the apical epithelial cells of conjunctiva and corneal surface secrete mucin for the aqueous mucus layer; the closer to the cornea, the higher the mucin amount (3). Under normal conditions, the blink reflex is performed 5–10 times/s, renewing tears, evenly distributing on the ocular surface, and promoting meibomian lipid release. The corneal nociceptive nerve innervates the blink reflex, originates from the ciliary nerve derived from the trigeminal node, and transmits through the cornea-scleral limbus to innervate the facial nucleus of orbicularis muscle and regulates the lacrimal gland or facial muscles. When the corneal sensitivity is reduced, resulting in less reflex tearing and fewer nerve impulses from the trigeminal nerve, the lacrimal gland secrete less (4). Dry eye is divided into anti-tear and evaporative types. Antitear dry eye is often associated with autoimmune diseases and is characterized by decreased lacrimal and other gland secretion. Evaporative dry eye and lack of meibomian gland lipid, blink rate reduces, main show is tear evaporation increased, but the amount of tear secretion is normal (5).

Inflammation is essential in dry eye pathogenesis; activating inflammatory pathways and releasing inflammatory factors lead to a vicious dry eye cycle (6). The dry eye clinical diagnosis must often understand the patient’s symptoms, environment, occupation, diet, contact lens usage, autoimmune disease, and surgery histories. Infrared imaging of the meibomian glands was given at times to patients to determine whether dry eyes were accompanied by meibomian gland dysfunction (7, 8). With the development of research on dry eye mechanism and diagnosis, the treatment approach should not be limited to artificial tears (ATs). Anti-inflammatory therapy seems to be a more suitable way to treat dry eyes. This review describes the dry eye inflammation mechanism, the associated inflammatory factors, and the anti-inflammatory treatment.

## 2 Dry eye and inflammation

### 2.1 Inflammatory cells and inflammatory factors

**Figure 1:**
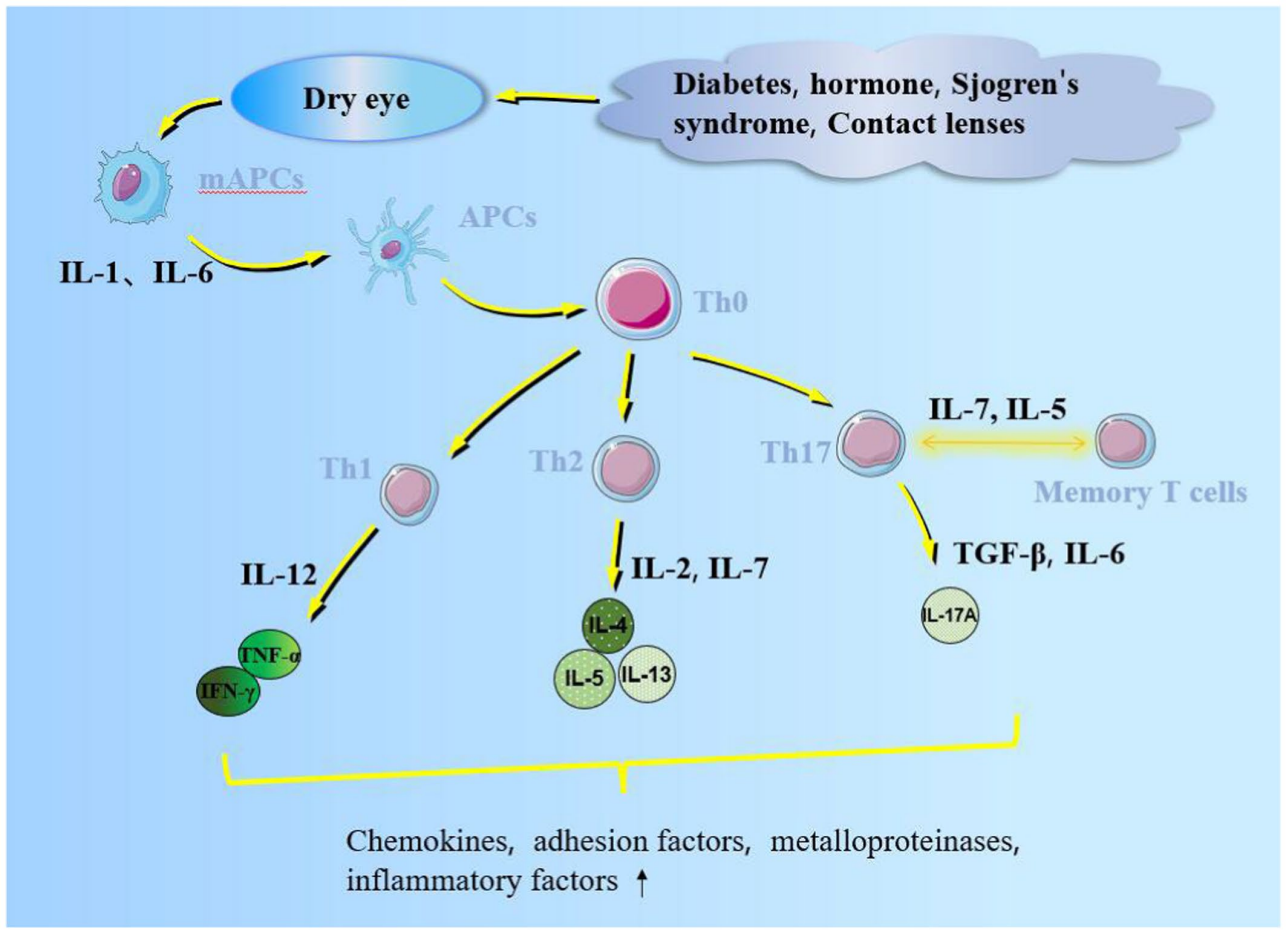
Inflammatory factors promote APC maturation. Mature APCs migrate through lymphatic vessels into regional lymph nodes with cytokines and chemokines. Additionally, it promotes naive T cells (Th 0) to form helper, memory, and regulatory T cells, which act on the ocular surface, secrete pro-inflammatory cytokines, and affect the corneal barrier function.

Inflammatory cells possess an immune role on the ocular surface, including neutrophils, macrophages, natural killer, dendritic, and T cells (9). Macrophages infiltrate the corneal epithelium of dry eye patients exhibiting higher levels than the control; therefore, macrophages may be related to inflammation grade and neovascularization (10). Natural killer cells induce epithelial cell death while secreting interferon-γ (IFN-γ), which triggers an inflammatory cascade (11). Activated Th0 migrates to dendritic cells, together with the corneal antigen between lymphatic endothelial and dendritic cells, interacting with each other (12). T cells are divided into CD4^+^/CD8^+^ T cells and CD4^+^ CD8^+^ double-positive T cells. CD4^+^ T cells include effector T cells: T helper 1 and 17 (Th1 and Th17) cells. CD8^+^ T lymphocytes, located in the secondary T cell zone of the thymus, are activated by antibodies on histocompatibility complex 1 (MHC-1) to form cytotoxic T lymphocytes with high CD57 content by antigen-presenting cells (APCs) (13, 14). Th17 cells are important in chronic diseases and disease relapse. Th-17 cell differentiation is initiated by the STAT3 signaling pathway activated by transforming growth factor-β (TGF-β) and interleukin (IL)-6 secreted by epithelial and dendritic cells. Activated Th17 secretes IL-17A, promotes nuclear factor-B (NF-κB) and mitogen-activated proteikinases (MAPK) signaling pathways to induce inflammation, stimulates metalloproteinase production, destroys the epithelial junction structure, and affects the corneal barrier function (15, 16); IL-12/23 can drive Th17 cells to affect ocular surface autoimmunity (17). Although IL-12/23 can stimulate the memory of Th17 cells, IL-7/15 can maintain this memory function for a long time and even lead to chronic inflammation (18). The memory effect of Th17 on dry eye could be alleviated by anti-IL-15 treatment (19). Th1 cells mediate cellular immunity; IL-4 promotes IL-12 secretion by APC cells in dry-eye patients, which induces Th1 cells to secrete IFN-γ and tumor necrosis factor-alpha (TNF-α) that enhance the endoplasmic reticulum stress, reduce Ca^2+^ concentration, and stimulate cholinergic agonist secretion to reduce mucin secretion by goblet cells. This inflammatory response promotes conjunctival goblet and lacrimal gland cell apoptosis (Figure 1) (20) and stimulates endothelial cells to secrete CXCL9/10/11 chemokines to aggregate inflammatory cells (21). Th2 cells mediate humoral immunity by secreting IL-4/5/13 due to the induction of WGATAR nucleotide consensus sequence (GATA-3) derived by IL-2/7 (22).

### 2.2 Inflammatory pathways

**Figure 2:**
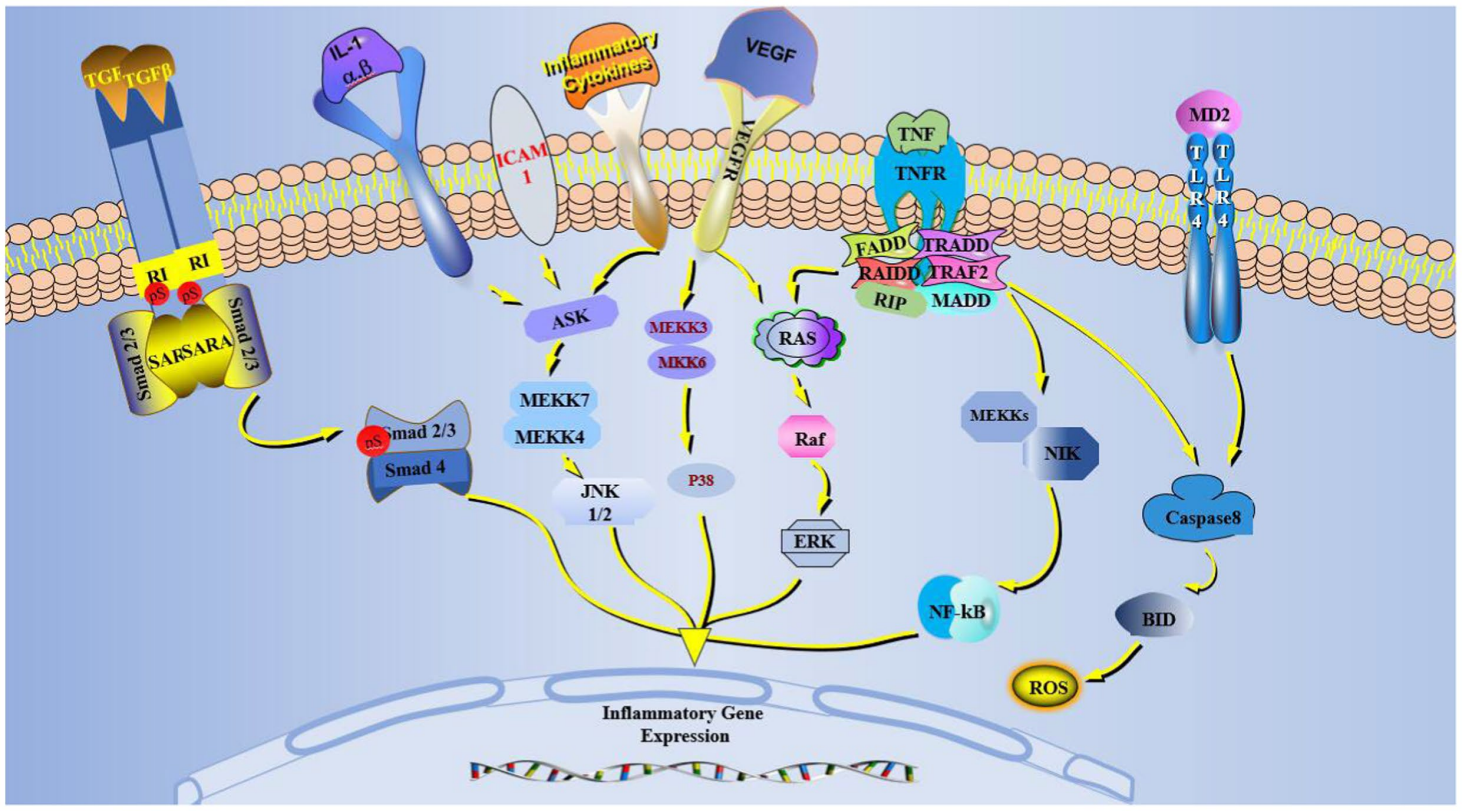
MAPK, VEGF, TGF-β, TNF, TLR-4 pathways in dry eye activated cells. Inflammation and VEGF activate MAPK, which generates JNK, P38, and ERK through ASK and Ras. TNF activates Ras and induces NF-κB production. TLR-4 activation induces ROS and inflammatory body production in cells. The TGF-β pathway binds and phosphorylates Smad2/3/4. The newly generated JNK, P38, ERK, NF-κB, and Smad2/3/4 in the cytoplasm enter the nucleus and affect the inflammatory gene expression.

The predisposing factors of dry eye include an inflammatory environment, tear hypertonicity, microtrauma, microorganisms, infection, and other stress, which trigger related inflammatory pathways. The common pathways included MAPK, NF-κB, and TGF-β, including three subtypes: JNK, ERK, and p38. The p38 pathway regulates Th1/2 cytokine secretion (23), activates NF-κβ downstream of the MAPK pathway, and translocates NF-κB to the nucleus to transcript pro-inflammatory factors (24). The inflammatory factors, TNF, IL, and matrix metalloproteinases (MMPs) expressions can activate the NF-κB pathway to amplify inflammatory effects (25). NF-κB can promote epithelial cells to assemble inflammasomes, activate caspase-1 after receiving inflammatory signals, and promote inflammatory factors IL-1β/18 expression (Figure 2) (26). These inflammatory factors can cause damage-associated molecular patterns (DAMPs) and activate pattern recognition receptors (PRRS) such as toll-like receptor 4 (TLR4). TLR4 can directly activate caspase-8 to promote inflammasome assembly further (Figure 1) (27). These inflammatory factors promote a corneal proteolytic enzyme, such as corneal MMP-3/9, which can hydrolyze the connexin between corneal cells and destroy the dense barrier structure of the cornea. Increased inflammatory factors and MMPs exacerbated the ocular surface state of high permeability, aggravating the dry eye symptoms and contributing to the chronic inflammatory reaction (28).

### 2.3 Inflammation and blood vessels

Blood vessels in normal cornea tend to be antiangiogenesis factor (Thrombospondin-1) (TSP-1); TSP-1 and endothelial cells of CD36 and CD47 inhibit angiogenesis (29). Vascular endothelium maintains the balance between internal and external blood vessels. Systemic diseases, such as Sjögren’s syndrome and diabetes mellitus, can cause systemic chronic inflammation, destroy the endothelium, and affect various organ structures. In endothelial dysfunction, after increased ROS, nitric oxide (NO) bioavailability is reduced, cell adhesion molecules are increased, and leukocyte infiltration is enhanced (30). Adhesion molecule intercellular adhesion molecule-1 (ICAM-1) in white blood cells, endothelial cells, and epithelial cell surface expression belongs to the immunoglobulin superfamily of albumin. The functionally associated antigen-1 (LFA-1) ligand rolls along the vessel wall and increases T cell adhesion to the endothelium in dry eye pathogenesis (31). The decrease of lacrimal gland secretion in diabetic dry eye patients is related to the lacrimal gland microvascular damage. In dry eye patients, the increased conjunctival blood vessel diameter increases the blood vessel density and blood flow rate, mainly related to cellular cytosine, NO, prostaglandins PGI2, PGE2, and histamine (32).

### 2.4 Inflammation and lymphatic vessels

Lymphatic vessels grow parallel in the limbus to form a network that maintains homeostasis by draining interstitial fluid and metabolites. During inflammation, blood vessels leak, generating increased interstitial fluid. Accordingly, lymphatic dilation allows more fluid and inflammatory cells to enter the lymphatic vessels to alleviate inflammation and maintain homeostasis (33). IL-1 induces IL-17 to promote vascular endothelial growth factor-D (VEGF-D) and VEGFR-3 expression in lymphatic vessels and increase lymphatic endothelial cell proliferation under dry stress (12). The VEGF and VEGF receptor families regulate lymphangiogenesis, among which VEGF-A/B mainly promote angiogenesis, while VEGF-C/D mainly promote lymphangiogenesis (34). VEGF-A/C are generated by tissues and then flow into lymph nodes, initiating lymph node production (35). VEGF-C/D activate ERK and AKT by binding to VEGFR3 to induce lymphatic migration and germination, and these lymphatic vessels migrate to the center of the cornea, which has a larger diameter of lymphatic vessels that grow into the cornea than the lymphatic vessels of the limbus (36, 37). The corneal lymphatic diameter and area decreased in VEGF-C-blocked dry-eye mice (38). VEGF-A expressed by B cells interacts with VEGFR-2, activating lymphatic proliferation without affecting new lymphatic vessel germination, besides inducing CD11b^+^ macrophage recruitment in the cornea injury site and promoting VEGF-A/C/D (39).

## 3 Common dry eye factors

### 3.1 Diabetes

Diabetes is an aggravating factor in dry eye patients. Due to neuropathy and metabolic disorders, diabetes may affect dry eye symptoms such as dryness, foreign body sensation, and burning sensation. The corneal epithelial layer of diabetic patients has high fragility, low thick bottom, and low cell density, weakening the barrier function (40). A high glucose environment promotes tissue protein glycosylation, AGE, and its receptor, which promotes the NF-kB signaling pathways activation and ROS (41). In the nucleus, NF-κB causes lacrimal gland cells to enter the polyol pathway. Glucose forms sorbitol under the action of aldose reductase; the accumulated sorbitol increases the lacrimal gland cell osmotic pressure, impairing the function of the lacrimal gland after cell edema (42). Oral aldose reductase inhibitors increase tear film evaporation time; therefore, insulin secretion affects meibomian gland function, lacrimal gland, and corneal epithelial proliferation (43). High glucose reduces the effect of trigeminal nerve nutrition, corneal nerve fiber density, length is reduced, patient blink rate drops, and it appears tear evaporation capacity increases. Panretinal laser therapy can affect corneal neuropathy and cause dry eye (44). In diabetic patients, meibomian gland duct obstruction, acinar atrophy, and surrounding inflammatory manifestations lead to meibomian gland dysfunction and dry eye (45). In the urea chain within induced diabetic rats, the lacrimal gland weight and volume are lower, while inflammation and related factors such as IL, TNF, and AGE were higher than in the control (46).

### 3.2 Sjögren’s syndrome

Sjögren’s syndrome is an autoimmune disease of the exocrine gland (such as tear ducts, salivary, sebaceous, and sweat glands). In women between 20–40 years old and with a high postmenopausal incidence, Sjögren’s autoantibody syndrome interferes with and damages a toadstool receptors, causing systemic symptoms (47). About 11% of dry eye patients can be diagnosed with Sjögren’s syndrome (48), which is characterized by swelling of lymphocytes in the lacrimal gland and conjunctiva, T lymphocyte attack with meibomian gland shedding, and mucin defense (49). In patients with Sjögren’s syndrome, antigen-antibody reaction may occur in the corneal stroma, inducing inflammation and causing collagen dissolution and corneal mechanobiology destruction (50). In patients with Sjögren’s syndrome complicated with dry eye, the positive rate of cornea staining is higher than in ordinary patients with dry eye.

The changes in corneal substromal nerve fibers in patients with Sjögren’s syndrome are debated; the chronic neuropathic pain in patients with dry eye in Sjögren’s syndrome may be related to decreased corneal substromal nerve. Li et al. (51) suggested that different disease courses or development stages may change corneal substromal nerve fibers differently. Ran et al. (52) suggested that corneal subbasal nerve fibers may be related to eye injury, tear production, and the response degree to such damaging stimuli.

### 3.3 Sex hormones

Women are more likely to suffer from dry eye due to sex hormones, as androgens promote lipid production while inhibited by estrogens (53). Androgen has been shown to have a regulatory role in the whole body of sebaceous glands, including the eye meibomian gland. The meibomian gland, as the largest sebaceous gland in the human body, produces 5α-reductase (54) and contains androgen receptors in the nucleus, which can convert testosterone into the active form of dihydrotestosterone, promoting DNA and protein synthesis in the lacrimal gland and increasing lacrimal gland secretion (55). For males, approximately 50% of the androgen production comes from the gonads; for women, almost all androgen originates from the adrenal glands, synthesized from adrenal steroid precursor (56). The probability of dry eye increases with age, especially in postmenopausal women, when the activity and content of the steroid precursor dehydroepiandrosterone (DHEA) in the adrenal gland and steroidogenic enzymes in the eye tissue decrease. Serum testosterone in premenopausal women had a negative correlation stability, and postmenopausal positively correlated. Accordingly, tears were positively correlated in premenopausal women; the opposite was true for postmenopausal (57). The local application seems to be better for the androgen treatment of dry eye than general replacement therapy. Estrogen has a certain anti-inflammatory effect; it can promote TNF-α and IL-6 expression and lymphocyte immune response. The decrease in sex hormones may cause dry eye symptoms in menopausal women. Conversely, for pregnant women in the third trimester, dry eye symptoms are caused by high estrogen and progesterone levels (58). Estrogen therapy for dry eye is controversial; for menopausal women, randomized controlled trials have shown that ATs combined with estradiol eye drops are more effective than ATs alone (59). Coksuer et al. (60) showed that postmenopausal women continued to take drospirenone 2 mg + estradiol 1 mg for six months, BUT and Schimer trial results improved. Although hormone therapy has a certain therapeutic effect on dry eye, long-term application may increase dry eye symptoms.

### 3.4 Contact lens

After using contact lenses, a more common term is end-of-day (EOD) symptoms, which refers to the dryness and discomfort of patients after removing contact lenses at night. Over half of people who wear contact lenses for a long time can be diagnosed with dry eye (61). The water content of contact lenses is inversely proportional to the prevalence of dry eye (62). Moreover, hydrogel contact lens material was more likely to lead to dry eye than silicone hydrogel material (63); the tear film lipid becomes thinner, and the osmolarity increases, which increases tear evaporation (64). Contact lenses of about 2 mm cover the conjunctival area; in a normal blink at the same time, contact lenses will move along the conjunctiva. Therefore, occasional contact lens breakage, leaving debris between the contact lens and the cornea, and other small debris may cause corneal and conjunctival wear and induce inflammation. IL-6/17A, MMP-9, ICAM-1, and granulocyte-macrophage colony stimulating factor (GM-CSF) levels increased in patients wearing contact lenses (65). The meibomian glands in contact lens wearers gradually shorten from the distal end and are more severely damaged than in ordinary dry eye patients. Conjunctival goblet cell density decreased after 3 months of contact lens wear (66, 67). Mild dry eye in contact lens wearers causes ocular surface inflammation and activation of dendritic cells in the cornea. The increased number of activated dendritic cells can decrease corneal basal nerve fibers. However, when dendritic cells are reduced, the corneal subbasal nerve fibers may increase due to regeneration (68). Prolactin, lipocalin-1, lactoferrin, and lysozyme are decreased in tears of dry eye patients. Compared with normal dry eye patients, prolactin content increased in those wearing contact lenses while the proline content decreased (69, 70).

## 4 Anti-inflammatory treatment of dry eye

### 4.1 Cyclosporine

Clinically, some patients with moderately severe dry eye drops use ring spore in ring spore essence, an isolate from the fungus immune modulators. The drug is positively charged due to its low water solubility and interacts with the negatively charged mucin in the tear film to improve its bioavailability as an oil-water emulsion (71). Cyclosporine is mainly metabolized in the liver of mammals and is not metabolized in the eye, where it mainly accumulates on the ocular surface and corneal epithelium. Continuous use of cyclosporine for 12 months hardly causes damage to the corneal endothelium (72). T cells can take up cyclosporine, which induces inflammation after being dropped on the ocular surface. By binding to cyclophilin in T cells, cyclosporine inhibits calcineurin, an enzyme required for T cell proliferation. However, due to the action of cyclosporine, T-cell proliferation is limited, and inflammatory factor formation is reduced (73).

Presently, 0.05%–0.1% cyclosporine eye drops are commonly used in the market. In dry eye patients without systemic diseases, 0.05% cyclosporine is recommended for 3 months. In patients with dry eye associated with systemic disease, 0.05% ring spore treatment is recommended for 6 months (74). In dry eye patients, 0.05% cyclosporine was used to take a conjunctival epithelial biopsy, revealing that conjunctival goblet cells and soluble mucin increased (75). In Mexican patients, 0.1% cyclosporine A was more effective in improving dry eyes, red eyes, and eye fatigue. The tear side effect is weak. Some studies have compared the effects of 0.05%, 0.1%, 0.2%, and 0.4% cyclosporine, indicating that 0.1% cyclosporine improved dry eye indexes (76). However, some dry eye patients may experience conjunctival congestion and tingling when using cyclosporine. Therefore, combining cyclosporine and other anti-inflammatory drugs in eye drops can reduce the drug-caused discomfort (77).

### 4.2 Corticosteroids

Cortical sterols and glucocorticoid receptor inhibition of inflammation factors promote lymphocyte apoptosis. The common corticosteroids in clinical practice include prednisone, dexamethasone, and fluorometholone. The stromal layer is hydrophilic because the corneal epithelium and endothelium are hydrophobic. Ethyl acetate, alcohols, and salts are commonly used as carriers of corticosteroids: acetic ester and alcohol derivatives to improve the fat-soluble carrier, phosphate, and hydrochloride to improve the water-soluble carrier (78). Methyl prednisone can reduce dry eye on MAPK pathway activation in mice, reducing inflammatory cytokine transcription in corneal epithelium to relieve dry eye inflammation (79). MMP-9, TNF-α, and IL-1β levels in tears of patients with dry eye were decreased after topical dexamethasone treatment. They also demonstrated that topical dexamethasone alleviated the hypertonic state of tears by inhibiting NF-κB activation through caspase-1 (80). Due to the inhibitory effect of dexamethasone on IL-1β, subsequent MMP-1 promoter activation is reduced, thereby inhibiting ERK and JNK phosphorylation (81). Dexamethasone controls MMP-8, which promotes corneal neutrophil infiltration and increases MMP-1/9/13 expression while impairing corneal healing in its absence. TIMPs are a two-way adjustment factor: TIMP-2 binds to MMP-2/14 to promote wound healing, and the opposite was observed for TIMP-1.

In conclusion, dexamethasone can treat the early inflammation of dry eye by reducing MMPs and TIMP-1, increasing MMP-8 transcription, and reducing neutrophil infiltration and inflammatory factor production (82). Long-term use of corticosteroids has side effects, such as increased intraocular pressure, infection aggravation, and posterior capsule opacity. Therefore, short-term initiation with soft steroids such as fluorometholone and loteprednol may be considered (83, 84).

### 4.3 Non-steroidal

Nonsteroidal antiinflammatory drugs (NSAIDs) are a safer alternative to corticosteroids for ocular inflammation control. Diclofenac sodium, bromfenac, and pranoprofen are common eye drops. NSAIDs inhibit cyclo-plus oxidase synthesis. COX, divided into COX-1/2, promotes arachidonic acid production of thromboxane, which induces inflammation (85). COX-2 was not present in normal cells but was sensitive to moderate to severe inflammation and chemical burns (86). NSAIDs and ATs were more effective in treating dry eyes than controls using ATs alone (87). Many think non-selective NSAID and COX-2 inhibition of COX-1 are simultaneously, but COX-1 inhibition affects the prostaglandin generated to promote corneal epithelium healing. Nimesulide, a selective non-steroidal anti-inflammatory drug, can preferentially inhibit COX-2, which is suitable for experimental animal dry eye rabbit models. In the dry eye rat model, tear osmolality increases, and extracellular ions are transferred into the cells to maintain stability, accelerating corneal cell apoptosis. During this period, the nuclear factor of T cell 5 (NFAT5) is activated, which regulates organic permeate formation and slows the increased intracellular ions (88). Topical administration of diclofenac sodium can stimulate NFAT5 production and reduce corneal epithelial damage without affecting tear volume (89). While NSAID drugs can alleviate the symptoms of dry eye patients, there is literature pointing out that NSAIDs can interfere with corneal defects in patients with corneal epithelium healing. NSAIDs can reduce the corneal sensitivity of ordinary individuals, and patients with dry eye, especially patients with Sjögren’s syndrome, should be carefully used. Severe cases may be accompanied by the possibility of corneal perforation (90).

### 4.4 Antibiotics

Currently, the antibiotics used in dry eye treatment include ofloxacin, Azithromycin, Doxycycline and minocycline tetracycline class. Local use of ofloxacin eye drops, although stability can be improved less in dry eye treatment (91). For dry eye caused by contact lens wear, 1.0% azithromycin eye solution can prolong the comfortable time of contact lens wearing and improve the dry eye degree (92). For dry eye with meibomian gland dysfunction, azithromycin can inhibit bacterial lipase formation, reduce meibomian gland lipid degradation by lipase, and promote the lipid content in tear film (93).

Azithromycin can stimulate meibomian gland cell differentiation, increase intracellular cholesterol, cholesterol lipid, phospholipid, and lysosome accumulation, promote new protein secretion, and improve dry eye (94, 95). As a broad spectrum of large ring lactone class antibiotic, azithromycin has a better permeability; it can block NF-κB activation, inhibiting inflammatory cytokines IL-6/8 (96). For dry eye patients with acne rosacea, tetracycline can reduce inflammatory factors IL-1α and TNF-α (97). Doxycycline inhibits MMP-9 formation by inhibiting TGF-β and activating the Smad and MAPK signaling pathways in corneal epithelial cells (98). Doxycycline can reduce the increased MMP-1/3/9/13 and inhibit JNK activation in human corneal epithelial cells under a hypertonic environment (99). Azithromycin and doxycycline can restore the carotenoid level in the tear film of patients with dry eyes. Therefore, the patients do not have dry eye symptoms and signs. Moreover, azithromycin seems more effective than doxycycline in improving meibomian gland secretion (100).

### 4.5 Autologous serum

Autologous blood serum contains epithelial growth factor (EGF), TGF-β, vitamin A, lysozyme, and fibronectin, which can improve epithelial cell density and promote the migration and repair of corneal epithelial cells (101, 102). For moderate dry eye, 20% autologous serum has a similar effect to 50% autologous serum; consequently, the recommended concentration is 20%, while for severe dry eye, 50% autologous serum is recommended (103). However, the treatment time is long, and it must be used for 3 months after the end of treatment, and the effect may be weakened with the extension of treatment time (104). Additionally, autologous serum should be sterile when prepared, used, and stored. Once contamination occurs, microorganisms will proliferate in the serum, likely aggravating the disease (105).

### 4.6 Other anti-inflammatory therapies

Hormone therapy for dry eye in postmenopausal women has been a research focus. The main mechanism of hormone therapy for dry eye is to reduce lacrimal gland inflammation, increase IgA levels, and promote meibomian gland secretion (106). Estrogen alone is more effective than estrogen combined with progesterone in improving tear and ocular surface symptoms in postmenopausal dry-eye women (107); especially for patients under the age of 50 menopause, the improvement effect is more significant (108). However, hormone therapy may increase intraocular pressure and even aggravate dry eye; this adverse risk may increase with age (109). IL-1 receptor antagonist (IL-1Ra, Anakinra) can effectively bind to IL-1R1, reduce inflammatory body production by macrophages, block the subsequent inflammatory response, and relieve pain in patients with dry eyes (110, 111). Meibomian gland massage, hot compress, and intense pulsed light (IPL) therapy are common for patients with meibomian gland dysfunction. Meibomian gland massage and hot compress can mainly open the obstructed meibomian gland mouth and remove the secretion accumulated in the meibomian gland (112). However, this moment of dredging on improving the meibomian gland dysfunction in patients with dry eye effect is short (113).

IPL therapy uses about 500–1,200 nm wavelength light in the eye and skin; it raises the skin temperature, induces superficial vascular coagulation, and while promoting meibomian gland secretion, it also attenuates the activity of inflammatory factors in the capillaries (114). IPL can target the pigment oxidase enzymes in cells that initiate the photobiomodulatory cycle, and the energy generated is used for cell repair (115). IPL can prolong the appearance of broken, repair rough corneal epithelium, and protect against mites (116). However, because the IPL energy strengthens when used, it may cause burns, the risk of bubbles, stabbing pain, and skin pigmentation (117). Mesenchymal stem cells (MSCs) can be transformed into various cells while regulating immune responses, repairing tissues, reducing CD4^+^ T cell infiltration, and reducing inflammation (118). Dry eye in the rat using bromine deoxyuridine labeled MSCs eye drops a week later, the conjunctival goblet cell increased, and tear volume and stability were improved (119).

The lymphocyte infiltration degree in the lacrimal gland of Sjögren syndrome dry eye mice decreased, and aquaporin 5 expression increased after intraperitoneal injection of bone marrow-derived MSCs (120). In addition to drug treatment of dry eye, dietary supplement research is widespread. Schirmer score and Schirmer score of dry eye patients who took royal jelly for 8 weeks were significantly improved. This may affect the potential mechanism of mitochondrial function in the lacrimal gland; AMPK phosphorylation improves the level of lachrymal energy to repair the tear (121, 122). Manuka honey, compared with normal honey, has higher polyphenol compounds and a large amount of methyl glyoxal, which makes it have better anti-inflammatory and antioxidant capacity (123), as it can improve ocular surface index, meibomian gland function and MMP-9 level (124).

Continuous supplementation of omega-3 fatty acids for 3 months reduced tear osmolality and increased tear film stability while reducing IL-17A levels (125). The ratio of ω-3 to ω-6 fatty acids determines whether inflammation occurs. Omega-6 fatty acids generated class arachidic acid has a pro-inflammatory role; omega-3 fatty acids were mostly eicosapentaenoic acid (EPA) and docosahexaenoic acid (DHA). EPA inhibits prostaglandin, leukotriene, and thrombinin production. DHA can be shortened and re-saturated to produce EPA and anti-inflammatory prostaglandins (126). In addition, added n-3 polyunsaturated fatty acids (n-3PUFA) and alpha lipoic acid (ALP) can increase tear secretion (127). Vitamin D can inhibit dendritic cell maturation, reduce inflammation, improve corneal endothelium, tear film index hyperosmolar state, and repair barrier function (128).

### 4.7 Other treatment

ATs are the most basic and conventional treatment for dry eyes. The different classifications of eye drugs can be divided into alternative and ocular surface wetting agents, more tears regulators three categories (129). Common moisturizer ingredients are carboxymethyl cellulose, carbomer, hyaluronic acid, betaine, sorbitol, glycerin, and salt ions. The structure of moisturizers is similar to mucin secreted by goblet cells, which can replenish water and improve tear film stability (19, 130). The common type of multi-action tear substitute is hypotonic ATs, which can treat dry eye by reducing the hyperosmolar condition. The hydroxyl and carboxyl groups of the drug can interact with the hydrogen bonds in the tear film (131). However, visual quality improves in some patients and deteriorates in others after using ATs (132, 133).

A dry eye with neuropathic pain can be relieved by nerve stimulation or gabapentin. Brinton et al. (134) implanted a nerve stimulator into the nasal submucosa of experimental rabbits. They gave a certain stimulation intensity and frequency every day, revealing that the volume of tears, the concentration of tear protein, and the content of lipids in the tears of rabbits increased, and the osmolality decreased. In 1927, the nasolacrimal reflex (NLR) was proposed; when the nasal mucosa is stimulated, it starts from the ophthalmic branch of trigeminal nerve, passes through the afferent nerve to anterior ethmoidal nerve, reaches the superior salivary nucleus (SSN) of midbrain and pons, and then passes through the parasympathetic branch of efferent nerve to meet the lacrimal nerve, innervating the lacrimal gland and accessory lacrimal gland, and controls tear production (135, 136).

Additionally, the electrical stimulation of tear ducts and lacrimal nerve, sieve before nerve, trigeminal nerve, and the cornea can increase the number of tears (137, 138). Gabapentin can bind to the α2δ subunit of presynaptic N-type voltage calcium channel, reduce excitatory neurotransmitter release, increase γ-aminobutyric acid (GABA) content, and relieve pain (139, 140). Gabapentin, for neurological encephalitis, diabetic neuropathy, and nerve after herpes zoster pain medication, showed the side effects of central nervous system suppression, such as dizziness, headache, and drowsiness (141). Vitamin A can enhance tear film stability and stimulate production (142). Ren et al. (143) demonstrated that oral vitamin B1 and mecobalamin in dry eye patients improved corneal nerve length, width, and reflectivity to accelerate epithelial cell repair. Additionally, maintaining eye hygiene and removing mites have certain benefits for dry eye treatment; using tea tree oil to remove mites can improve dry eye symptoms (144).

## 5 Summary

Dry eye is a common disease that is usually managed using ATs. The main way to detect dry eye inflammation is to measure the cytokines in tears, such as anti-inflammatory cytokines IL-1b, IL-8, IL-17A, IFN-g, TNF-a or anti-inflammatory factor IL-10. The commonly used tear collection methods include Schirmer test paper collection and glass microcapillary collection. The difference is that the latter is suitable for the detection of a wider range of proteins in tears, while the former is more suitable for the detection of inflammatory factors (145, 146). With the wide application of new examination techniques in clinical practice, the number of inflammatory cells and dendritic cells in the corneal stroma of dry eye patients has increased by confocal microscopy. Currently widely used is the HRT3 confocal microscope produced by Heidelberg, Germany, which has a magnification of 800 times. The lateral resolution was 1–2 μm and the axial resolution was 5–10 μm. The field of view is smaller because of the larger magnification. For examination, topical anesthesia was performed in advance and the microscope objective was covered with a polymethyl methacrylate cap. The instrument probe was placed in contact with the corneal surface, and the images were collected by manually adjusting the controller until the corneal substromal nerve fiber layer could be clearly seen (147, 148).

With primary diseases such as Sjögren’s syndrome, diabetes patients should be actively treating the primary disease. A dry eye with inflammation produces various inflammatory factors (Table 1). Therefore, anti-inflammatory treatment combined with ATs seems more effective in improving dry eye mechanisms. Non-steroidal anti-inflammatory drugs can be used in patients with long-term contact lens wearing because of papillae and follicles in the palpebral conjunctiva. 0.1% cyclosporine is not recommended for moderate dry eyes because it irritates the eye, and 0.05% cyclosporine can be added first. Using corticosteroids can be anti-inflammatory and improve dry eye for postoperative dry eye. For dry eyes associated with meibomian gland dysfunction, and with the meibomian gland massage and hot compress, strong pulse therapy appears to relieve the discomfort of the patient instantly and reduce the tarsal cyst and stye.

**Table 1:** Inflammatory factors associated with dry eye.

Cytokines	Produce cells	Function
IL-12/23	Dendritic cell/macrophage/monocyte activation	Stimulate Th17 cell transformation into memory Th17 cells
IL-1/6	Macrophages and fibroblasts	Promotes APC maturation
TGF-β	Epithelial and dendritic cells	Initiates Th-17 cell differentiation
IL-7/15	Catecholaminergic and peripheral T cells	Maintain the memory function of Th17 cells for a long time
IFN-γ and TNF-α	Th1	Enhance endoplasmic reticulum stress; decrease Ca^2+^ concentration; stimulate cholinergic agonist secretion; reduce mucin secretion by goblet cells; stimulate endothelial cells to secrete the chemokine CXCL9/10/11; promote conjunctival goblet and lacrimal gland cell apoptosis
IL-2/7	Th1	The nucleotide consensus sequence is driving WGATAR (GATA-3). Induce IL-4/5/13 secretion by Th2 cells
IL-4	Th2	Promotes IL-12 secretion by APCs cells, the initiating signal to secrete IFN-γ and TNF-α by Th1 cells
IL-4/13	Th2	Induce B cell differentiation into IgE/G1/G4
IL-5	Th2	Promote eosinophils
IL-17	Th17	Stimulates Treg function and increases MMPs; promotes lymphatic generated and B cell proliferation to promote ocular surface disease; stimulates MMP-3/9 production

In treating meibomian glands, steroid hormones such as dexamethasone, Pyridostigmine and antibiotic azithromycin can achieve twice the effect with half the effort. Compared with other anti-inflammatory drugs, the autologous serum has more immunoreactive ingredients and seems better for use in concurrent keratitis under conditions of protection from microbial infection. Although dry eye is more common in elderly female patients, it is necessary to monitor sex hormone levels while regularly reviewing dry eye. Therefore, it was suitable for endocrine disorder patients. Diet therapy is closer to life, and it is appropriate to eat some royal jelly and manuka honey, which contain omega-3 fatty acids and vitamins A/B1/D, for some patients are more likely to accept (Table 2).

**Table 2:** Anti-inflammatory drugs for dry eyes.

Drugs	Mechanism	Disadvantages
Cyclosporine	Inhibit calcineurin	Conjunctival congestion and tingling of the eyes
Cortical sterols	Reduce MAPK pathway and NF-κB activation	Increased intraocular pressure; aggravated infection; posterior capsule opacification
Non-steroidal	Inhibit cyclooxygenase synthesis	Decrease corneal sensitivity
Antibiotics	Inhibit bacterial lipase formation; stimulate meibomian gland cell differentiation; increase intracellular lipid accumulation; activate NF-κB was blocked	Fewer applications
Autologous serum	Increase epithelial cell density; promote corneal epithelial cell migration and repair; increase goblet cell count	Easy to contaminate
Hormone therapy	Reduce lacrimal gland inflammation; increase IgA level; promote meibomian gland secretion	Increased intraocular pressure; older patients had poor outcomes
IL-1 receptor antagonist	Combined with IL-1R1, it reduced inflammatory body production by macrophages	New drugs are rarely used
Meibomian gland massage and hot compress	Dredge the meibomian gland orifice; remove the secretion accumulated in the meibomian gland	Improvement is short-lived
Intense pulsed light	Induce superficial vascular coagulation; reduce inflammatory factor activity in capillaries; produce energy to repair cells	Risk of burns, bubbles, tingling, skin pigmentation
MSCs	Regulate the immune response; repair tissue; reduce CD4^+^ T cell infiltration; increase aquaporin 5	High cost
Royal jelly	Affect mitochondrial function and AMPK phosphorylation in the lacrimal gland	Fewer studies
Manuka honey	The structure contains polyphenol compounds and a large amount of methylglyoxal, anti-inflammatory, and anti-oxidation	The specific dosage is difficult to determine
Fatty acids	Inhibit prostaglandins, leukotrienes, and thrombinins, shortening and re-saturating to produce EPA and anti-inflammatory prostaglandins	The specific dosage is difficult to determine
Vitamins	Inhibit dendritic cell maturate; improve corneal endothelium and tear film index hyperosmolar state	It is more suitable for patients with vitamin deficiency
ATs	Rehydration, reduction of hypertonic state, and reduction of evaporation	Decreased visual quality
Stimulation of nerves	Lacrimal secretion is stimulated via various structures of the nasolacrimal reflex	Human experiments are more difficult to perform
Gabapentin	Reduce excitatory neurotransmitter release; increase GABA content; relieve pain	Central nervous system depression, such as dizziness, headache, and drowsiness

Various anti-inflammatory drugs correspond to dry eyes with different symptoms and signs. As for the dosage and time of medication, rheumatic diseases should be recommended if the patient’s tear score is deficient at the first visit. In terms of medication, ATs were given. A conventional dose of anti-inflammatory drugs was given according to the signs, and the patient was advised to review within a certain period. According to the review of dry eye score, intraocular pressure is considered, and an anti-inflammatory drug is used. Anti-inflammatory drugs should not be given without a time limit and should be discontinued during ongoing patient review. The information should include the patient’s age, gender, lifestyle, job characteristics, diet, drug reaction conditions, and acceptance. In clinical practice, patients with dry eye who often visit again feel that their ocular discomfort has not been significantly relieved. Therefore, we should ask about the patient’s medical history in detail, such as using regular medication and the work and living environment status. After careful physical examination, the regimen should be re-determined, and a combination of antibiotics may be considered.

There are many anti-inflammatory studies on dry eye treatment, but a few studies on the course and dosage. This may be related to the fact that dry eye is a complex multifactorial disease. Conventional anti-inflammatory drugs, such as non-steroidal drugs and corticosteroids, are routinely used in clinical practice. Drugs that inhibit inflammatory pathways and alleviate inflammatory factors are not widely used due to the lack of extensive clinical trials. In conclusion, dry eye treatment should be multiple and comprehensive, particularly combined anti-inflammatory drugs and ATs. For the future anti-inflammatory treatment of dry eye, we continue to work hard to clarify the dosage and the development of new drugs (149).

## Author contributions

LC: Writing – original draft. CW: Writing – review & editing. HZ: Funding acquisition, Methodology, Project administration, Resources, Writing – review & editing.
